# Sociodemographic correlates of urine culture test utilization in Calgary, Alberta

**DOI:** 10.1186/s12894-018-0315-x

**Published:** 2018-01-08

**Authors:** Thomas P. Griener, Christopher Naugler, Wilson W. Chan, Deirdre L. Church

**Affiliations:** 1 Department of Pathology and Laboratory Medicine, University of Calgary, Calgary, AB Canada; 2 Department of Family Medicine, University of Calgary, Calgary, AB Canada; 3 Division of Microbiology, Department of Pathology and Laboratory Medicine, University of Calgary, Calgary, AB Canada; 4 Department of Medicine, University of Calgary, Calgary, AB Canada; 5 1W-410, Diagnostic and Scientific Centre, 9-3535 Research Road NW, Calgary, AB T2L 2K8 Canada

## Abstract

**Background:**

Many clinical practice guidelines encourage diagnosis and empiric treatment of lower urinary tract infection without laboratory investigation; however, urine culture testing remains one of the largest volume tests in the clinical microbiology laboratory. In this study, we sought to determine if there were specific patient groups to which increased testing was directed. To do so, we combined laboratory data on testing rates with Census Canada sociodemographic data.

**Methods:**

Urine culture testing data was obtained from the Calgary Laboratory Services information system for 2011. We examined all census dissemination areas within the city of Calgary and, for each area, testing rates were determined for age and gender cohorts. We then compared these testing rates to sociodemographic factors obtained from Census Canada and used Poisson regression and generalized estimating equations to test associations between testing rates and sociodemographic variables.

**Results:**

Per capita urine culture testing is increasing in Calgary. For 2011, 100,901 individuals (9.2% of all people) received urine cultures and were included in this analysis. The majority of cultures were received from the community (67.9%). Substantial differences in rate of testing were observed across the city. Most notably, urine culture testing was drastically lower in areas of high (≥ $100000) household income (RR = 0.07, *p* < 0.0001) and higher employment rate (RR = 0.36, *p* < 0.0001). Aboriginal – First Nations status (RR = 0.29, *p* = 0.0008) and Chinese visible minority (RR = 0.67, *p* = 0.0005) were also associated with decreased testing. Recent immigration and visible minority status of South Asian, Filipino or Black were not significant predictors of urine culture testing. Females were more likely to be tested than males (RR = 2.58, *p* < 0.0001) and individuals aged 15–39 were the most likely to be tested (RR = 1.69, *p* < 0.0001).

**Conclusions:**

Considerable differences exist in urine culture testing across Calgary and these are associated with a number of sociodemographic factors. In particular, areas of lower socioeconomic standing had significantly increased rates of testing. These observations highlight specific groups that should be targeted to improve healthcare delivery and, in turn, enhance laboratory utilization.

## Background

Lower urinary tract infection (LUTI, cystitis) can be reliably diagnosed without laboratory investigation based on a focused history of urinary symptoms (frequency, urgency and dysuria) in the absence of urethral discharge or vaginal irritation. Adult women with symptomatic, uncomplicated LUTI should receive short-course empiric antibiotic therapy and do not require urinalysis or urine culture for diagnostic confirmation of bacteriuria or pyuria. Numerous clinical practice guidelines support this and encourage the use of urine culture testing in the adult population primarily for bacterial identification and antibiotic sensitivity testing in patients not responding to therapy or with recurrent disease [1–4]. However, urine culture testing is necessary in all cases of upper urinary tract infection (pyelonephritis) and screening cultures for asymptomatic bacteriuria is also indicated in pregnancy and for those undergoing urologic procedure where bleeding is anticipated [5].

These recommendations are largely based on the following important considerations. Bacteriuria is not itself a disease state and is typically not an indication for antibiotic therapy [5]. Asymptomatic bacteriuria is particularly common in the elderly population, with prevalence estimates of 3.6–19% in those aged 70 or over and living in the community [5, 6]. In nursing home residents, prevalence is estimated at up to 50%. It is not associated with increased morbidity or mortality [7]. Guidelines from the Infectious Disease Society of America (IDSA) and Choosing Wisely initiatives emphasize that even in the presence of pyuria, asymptomatic bacteriuria does not normally require treatment [5, 8]. The clinical significance of bacteriuria is therefore defined by patient signs and symptoms. Furthermore, the majority of urine specimens submitted for culture are the midstream portion of voided urine and culture results from these minimally-invasive collections have poor specificity, with a false positive rate of over 40% when compared to suprapubic needle aspiration or catheterization as gold standard [3, 9]. A positive urine culture therefore does not prove true bacteriuria, and certainly does not prove the presence of a urinary tract infection. Several strategies have been adopted by clinical microbiology laboratories to minimize reporting of contaminated specimens. The most common method is reporting of quantitative culture, for which there are established thresholds of bacterial quantity which define clinical significance [3, 5, 10]. However, in symptomatic patients, colony count thresholds likely underestimate the clinical significance of certain potential urinary pathogens while overestimating others [11]. For example, *Escherichia coli* can be associated with symptomatic disease even when isolated at quantities that are orders of magnitude below these thresholds. These combined factors minimize the value of urine culture results in both asymptomatic and symptomatic individuals.

Despite these problems, urine culture remains an important test that directs antibiotic therapy and further investigations in certain clinical settings. However, there is substantial practice variation in the use of diagnostic tests in LUTI and urine culture remains one of the highest volume tests in clinical microbiology laboratories [12]. Inappropriate testing is not without consequence as positive urine culture results likely drive unnecessary antibiotic treatment and contribute to rising rates of antibiotic resistance and other adverse events associated with antibiotic usage including *Clostridium difficile* infection [13–18]. We undertook the current ecological study to determine what variation in urine cultures testing existed across Calgary, Alberta and to determine what sociodemographic factors were associated with increased urine culture testing. Such information will allow targeted investigation into causes of potential over-utilization and more focused intervention strategies.

## Methods

The study protocol was approved by the University of Calgary Conjoint Health Review Ethics Board and a waiver of consent was granted (ID#REB15–0629).

This observational study combined laboratory data with variables obtained from the 2011 Census Canada Canadian Household Survey, the most recent such survey at the time this study was completed. The study population consisted of all individuals within Calgary, Alberta who underwent urine culture testing between January 1, 2011 and December 31, 2011. All urine culture testing at Calgary Laboratory Services (CLS) is performed as part of routine patient care and samples are analyzed in a single laboratory. CLS is the only testing laboratory in Calgary, Alberta and data from the CLS laboratory information system (LIS) therefore represents a comprehensive view of urine culture testing in the entire city (2011 population 1.1 million). In addition to data for the 2011 year, the number of monthly urine cultures collected from the LIS for each month from January 2010 to December 2013 in order to measure trends in urine culture ordering.

Each patient was included only once in the analysis to avoid pseudo-replication. For each test record, the following information was extracted from the laboratory information system: urine culture result, age, sex and health care number. Health care number was used as a linking variable to determine subject postal codes from an Alberta Health database, which were then converted to their corresponding geographic coordinates and census dissemination areas (CDA). CDAs are the smallest geographic groupings used for Canadian census data and contain 300–700 individuals. The data was then permanently de-identified. We included only individuals living within the City of Calgary.

For each CDA, the following sociodemographic variables were linked from the Canadian Household Survey: median household income ≥ $CDN 100,000 (overall median household income in Calgary for 2011 was $93,410), level of education (percent with university education), percent of individuals of Aboriginal - First Nations descent (North American Indian, as defined by Statistics Canada [19]), percent of individuals immigrating to Canada within the past 5 years and percent of individuals of Chinese, South Asian (primarily Indian, Pakistani and Sri Lankan), Filipino or Black visible minority status (the four most common statuses in Calgary, as defined by Statistics Canada [20]). We then examined these data-sets for associations between these sociodemographic variables and urine culture testing rate.

**Fig. 1:**
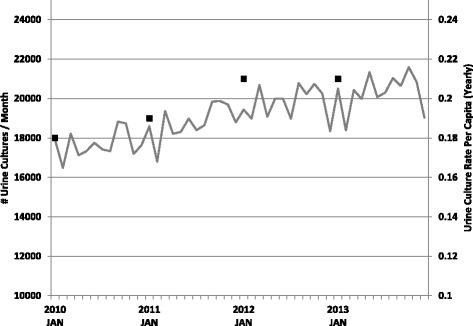
Urine culture testing at CLS from January 1, 2010 to December 31, 2013 presented as the number of urine culture tests per month (grey line) and the yearly per capita urine culture test rate (black squares)

For per capita testing rate (Fig. 1), the total number of urine culture tests for each year was divided by the total Calgary population as reported by the annual City of Calgary Civic census (http://www.calgary.ca). To calculate the percent of testing within each age and gender group (Table 1), the total number of individuals within each group who received at least one urine culture was divided by the total population within that group in Calgary according to the 2011 Canadian census. To calculate the testing frequency for the age and gender group in each CDA, the number of individuals that received testing from that group was divided by the total number of that age group in the dissemination area. For visual representation of the data, the values were then plotted onto a dissemination area map for Calgary using the ArcGIS v9.3 geo-mapping software (Environmental System Research Institute, Redlands, California). This software tool uses Getis-Ord Gi^*^ statistic to produce z-scores and identify statistically significant hot (increased testing) and cold (decreased testing) spots depending on how many standard deviations the data is removed from the mean [21]. Statistical inference regarding sociodemographic variables associated with testing rate was performed using the generalized estimating equations version of Poisson regression in SAS v9.4. The reported relative risks refer to the independent contribution of each variable with the other categorical variables (age, gender, group) held constant at an arbitrary reference value. The differences in testing rates are reported as relative risk (RR) for the independent contribution of that variable to the analysis and results were considered statistically significant at a *p*-value of 0.05.

**Table 1:** Frequency of Urine culture testing in Calgary, Alberta for 2011

	Number of Tests	Individuals Tested	Total Population	Percent Tested
Total	219,015	100,901	1,096,830	9.2%
Male	40,294	23,009	547,475	4.2%
<15	4999	3752	100,450	3.7%
15-39	6046	4410	208,840	2.1%
40-49	4083	2653	86,935	3.1%
50-59	6130	3407	77,460	4.4%
60-69	5870	3129	41,920	7.4%
≥70	13,166	5658	31,875	17.8%
Female	132,388	77,892	549,360	14.2%
<15	10,292	7105	95,970	7.4%
15-39	53,888	34,282	205,995	16.6%
40-49	14,449	9393	84,950	11.1%
50-59	14,110	8716	75,415	11.6%
60-69	11,755	6437	43,075	15.0%
≥70	27,894	11,959	43,940	27.2%

## Results

Increased testing volumes and per capita testing rates have been reported in most laboratory divisions [22]. Urine culture testing, which is the highest volume microbiology test at CLS, has seen a similar rise that exceeds Calgary’s population growth (Fig. 1). Linear regression analysis of this data shows statistically significant increases in test volumes (*R*^2^ = 0.68, *P* < 0.001) and year over year per capita test rates (*R*^2^ = 0.96, *P* = 0.002).

Data on 225,473 urine culture results were available in our LIS for 2011, which represented 133,464 individuals who underwent urine culture testing. After excluding individuals where no postal code was available and those with postal codes outside the city of Calgary, 100,901 individuals remained and were included in our study. The majority of specimens were received from patients in the community (67.9%) and the emergency department (19.3%), with the remainder from nursing homes (2.3%), inpatients (5.3%) and various outpatient settings including pre-admission clinics and subspecialty clinics (collectively 5.2%). Overall, 9.2% of individuals in the city of Calgary were tested during the study period, including 4.2% of males and 14.2% of females (Table 1). For males and females, the highest testing rate was in individuals ≥70 years (17.8% and 27.2%, respectively), however the greatest number of specimens were received from females aged 15–39. In part, this increased testing rate is likely accounted for by screening for asymptomatic bacteriuria in pregnancy but such clinical information was not available in our study.

**Fig. 2:**
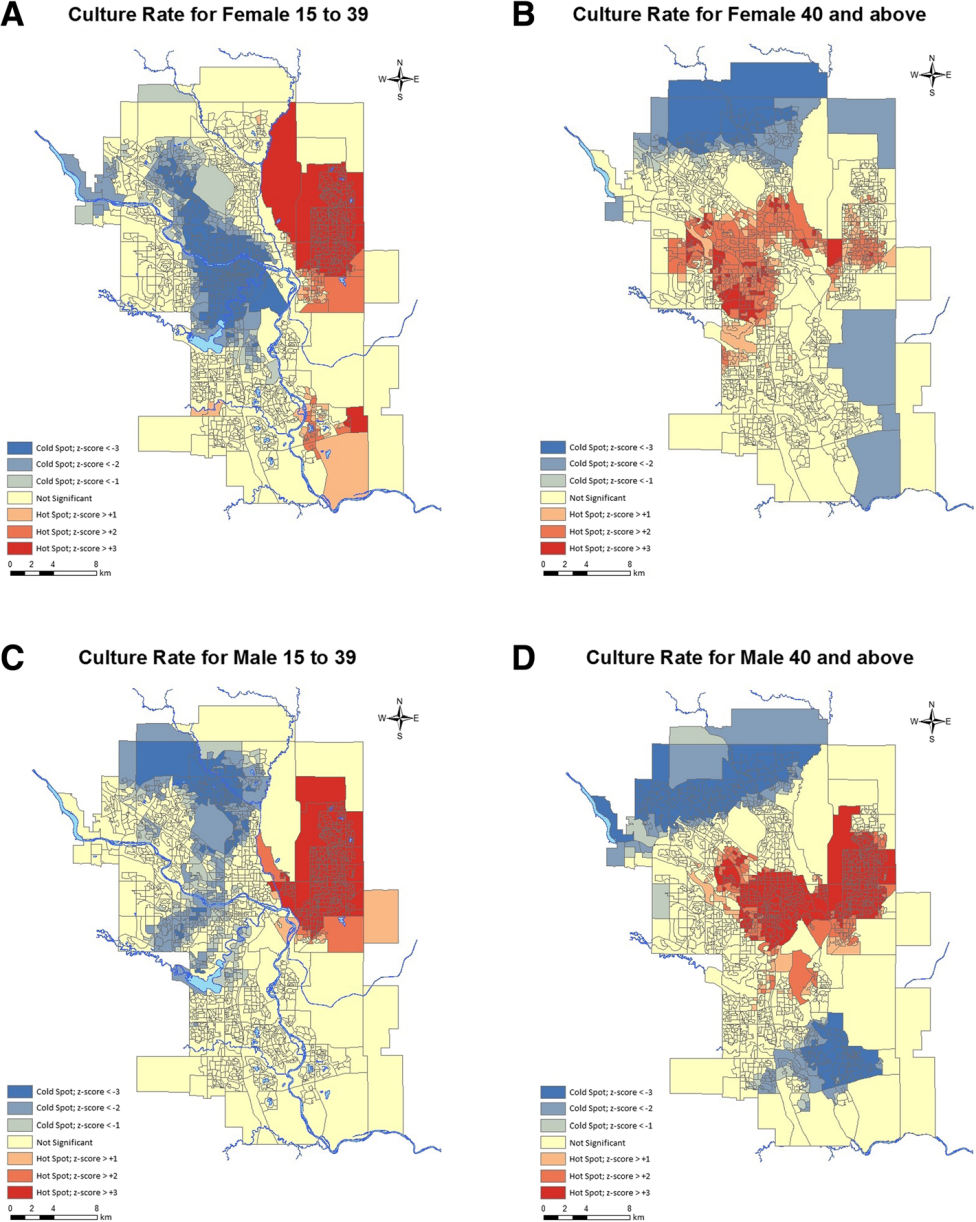
Hotspot maps representing the frequency of urine culture test ordering in four age/gender groups. The testing rate (individuals tested / total individuals in dissemination area) is represented by the number of standard deviations (z-score) it is removed from the mean (yellow/beige) in the positive (red, increased testing) and negative (blue, decreased testing) direction. Culture rates are shown for Females aged 15–39 (**a**), Females aged ≥40 (**b**), Males aged 15–39 (**c**), Males aged ≥40 (**d**). Maps generated using ArcGIS v9.3 geo-mapping software

The ArcGIS hot spot analysis mapping illustrates significant differences in screening rates across the city (Fig. 2). Significantly increased testing is observed in the inner city and northeast quadrants of the city. The median urine culture testing rates among neighbourhoods was 9.1% with 7.5% and 10.9% as the lower and upper quartiles, respectively.

The association between sociodemographic variables and urine culture test rates are shown in Table 2 and many inequities are present. The regression model showed that females were significantly more likely to be tested than males (RR = 2.58, *p* < 0.0001). As seen in Table 2, individuals aged 15–39 were the most likely to be tested (RR = 1.69, *p* < 0.0001), followed by those ≥70 years of age (analysis control, RR = 1.0). Decreased urine culture testing was associated with higher (≥ $100000) household income (RR = 0.07, *p* < 0.0001) and higher employment rate (RR = 0.36, *p* < 0.0001). No association between university education and testing was detected. Aboriginal – First Nations individuals (RR = 0.29, *p* = 0.0008) and individuals of Chinese descent (RR = 0.672, *p* = 0.0005) were also less likely to be tested. Interestingly, recent immigration (≤ 5 years), South Asian descent, Filipino descent or Black visible minority status were not significant predictors of urine culture testing.

**Table 2:** Sociodemographic variables and Urine culture testing rates in Calgary, Alberta for 2011

Socio-demographic Variable	Relative Risk (RR)	RR 95% Confidence Interval		Parameter Estimate	*P*-value
Female	2.583	2.539	2.629	0.9491	<0.0001
Male^a^	1.000	Reference		Reference	Reference
Age < 15	0.586	0.548	0.627	−0.534	<0.0001
Age 15–39	1.689	1.587	1.797	0.524	<0.0001
Age 40–49	0.624	0.587	0.663	−0.472	<0.0001
Age 50–59	0.660	0.623	0.700	−0.415	<0.0001
Age 60–69	0.550	0.520	0.581	−0.599	<0.0001
Age ≥ 70^b^	1.000	Reference		Reference	Reference
Median Household Income ≥$100,000	0.074	0.038	0.147	−2.600	<0.0001
Employment Rate	0.367	0.251	0.537	−1.002	<0.0001
University Education	1.091	0.864	1.377	0.087	0.464
Recent Immigrant (≤ 5 years)	0.678	0.440	1.044	−0.389	0.077
Aboriginal – First Nations	0.289	0.140	0.596	−1.240	0.0008
Chinese	0.672	0.536	0.842	−0.398	0.0005
South Asian	0.924	0.779	1.097	−0.079	0.3656
Filipino	0.791	0.563	1.111	−0.235	0.1761
Visible Minority - Black	0.878	0.392	1.965	−0.130	0.7515

^a^Males were used as reference for females

^b^Age group ≥70 was used as a reference for the other age groups

## Discussion

Our data reveal a drastically lower rate of urine culture testing with higher household income (≥ $100000) and higher employment rate. In previous studies, we demonstrated that these factors were associated with increased 25-hydroxyvitamin D and prostate specific antigen (PSA) testing and we hypothesized that this was possibly due to greater access to health care and patient-requested testing [23, 24]. The differences in urine culture testing identified in the present study appears contrary to that hypothesis. Why such drastic differences in urine testing rates exist between higher and lower socioeconomic status groups is unclear. One possibility is that the testing differences are warranted and reflect increased rates of LUTI, complicated urinary tract infection or antibiotic resistance in lower income earners. Previous studies have demonstrated a modest association between lower household income and community-acquired urinary tract infections and asymptomatic bacteriuria [25–27]. Increased incidence of other genitourinary tract infections causing similar symptoms, such as *Neisseria gonorrhoeae* or *Chlamydia trachomatis* urethritis/vulvovaginitis, could result in greater urine culture testing as well. In support of this, an earlier study showed a similar association between lower socioeconomic indicators and prevalence of these infections in Calgary [28].

It is also possible that discrepancies in urine culture ordering are unrelated to genitourinary tract infection, but instead result from differences in the prevalence of diseases with overlapping clinical presentation. Irritative lower urinary tract symptoms (urinary urgency, incontinence and frequency) consistent with overactive bladder syndrome (OAB) and/or benign prostatic hyperplasia (BPH) increase with age and may elicit urine culture testing [29]. Previous studies have demonstrated a relationship between higher income, higher levels of education and employment status and fewer of these symptoms in both women and men [30, 31]. The authors of these studies speculated that the reduced symptoms were the result of regular health check-ups, earlier recognition of symptoms and prior treatment. This could also explain our findings as patients of lower socioeconomic status may have more persistent urinary symptoms and have more urine cultures performed while not receiving appropriate therapy for these non-infectious conditions as a result of less consistent healthcare contact. Further studies are needed to determine whether this is indeed the case, as appropriate intervention would substantially improve patient care while also reducing microbiologic testing.

Concern regarding antibiotic resistant organisms could also drive increased urine culture utilization and clinical guidelines support the use of urine cultures in these instances. Resistant rates vary throughout the world; however, the Study for Monitoring Antimicrobial Resistance Trends (SMART) has established that the highest levels of antimicrobial resistance exist in the Asia-Pacific region where as many as 28.2% of LUTI pathogens possess extended spectrum β-lactamases [32]. South Asian, Chinese and Filipino people are strongly represented in Calgary’s visible minority population, and increased urine culture test rates would be expected if there were concerns about antimicrobial resistance. In fact, our data reveal the opposite trend with Chinese ethnicity associated with a statistically significant decrease in testing, and no relationship between test rate and recent immigration, South Asian or Filipino ethnicity. The cause of this decreased testing is unclear, but could represent barriers preventing health care access. Given the risk of resistance, this is a potential cause for concern.

The main strength of this study is the large sample of patients and, because our laboratory performs testing for all of Calgary, the data presented herein represents a complete view of urine culture testing on the adult (>15) population of the city. However, the limitations of this study must be recognized when interpreting these results. Because this study was retrospective and involved a very large number of patients, we were unable to collect clinical information or assess other concurrent laboratory testing (such as urinalysis or testing for sexually transmitted infection). As a result, we cannot ascertain the clinical appropriateness of urine culture testing. However, the variability in test ordering across the city without clear explanation is highly suggestive of inappropriate utilization. Patients with underlying disease may necessitate increased urine culture testing and such patients were certainly included in this study. We attempted to minimize the impact of such patients by counting a single urine culture per individual. As well, as this is an ecological study, inferences are made based on group level variables that may not accurately represent individual level variables. Some of the differences identified may be the result of confounding variables that are not captured in this study. It is also unknown what degree of urine testing is driven by patient or physician characteristics (generalist versus specialist practice, experience, location of training, etc.) and physician variables were not controlled for in the current study. Despite these deficits, we have identified several sociodemographic groups with significantly increased rates of testing and have demonstrated substantial variation in urine culture utilization across Calgary.

Further work will be to expand these studies to investigate for mismatch between urine culture ordering practices and clinical utility. Assessment of urine culture positivity rates and antibiotic resistance rates and how they align with the test utilization rates presented in the current study will provide useful information to direct intervention strategies to improve appropriate urine culture usage.

## Conclusions

Despite clinical practice guidelines recommending limited use of urine culture in diagnosing LUTI, we have shown that test rates continue to increase in Calgary and that substantial heterogeneity exists in test utilization across the city. We have also identified several patient groups with greatly increased or decreased test rates that may be indicative of inappropriate test utilization. Future investigations will now be focused on these patient groups to ascertain specific explanations for the potential over-utilization observed herein.

## Abbreviations

CDA: Census dissemination area
CLS: Calgary Laboratory Services
IDSA: Infectious Disease Society of America
LIS: Laboratory Information System
LUTI: Lower urinary tract infection
RR: Relative Risk
